# Surgical Treatment of Severe Aortic Stenosis: Sutureless Versus Stented Bioprosthetic Aortic Valve Replacement

**DOI:** 10.3390/jcm14165906

**Published:** 2025-08-21

**Authors:** Alessandro Ricasoli, Carmelo Mignosa, Salvatore Lentini, Laura Asta, Adriana Sbrigata, Claudia Altieri, Calogera Pisano

**Affiliations:** 1 Cardiac Surgery Unit, University of Catania, 95124 Catania, Italy; alessandro.ricasoli@gmail.com (A.R.); carmelo.mignosa@gmail.com (C.M.); eiko251961@gmail.com (S.L.); 2 Cardiac Surgery Unit, Department of Neuroscience, Imaging and Clinical Sciences, University “G.d’Annunzio” Chieti-Pescara, 66100 Chieti, Italy; 3 Cardiac Surgery Unit, Department of Precision Medicine in Medical Surgical and Critical Area (Me.Pre.C.C.), University of Palermo, 90134 Palermo, Italy; adriana.sbrigata@gmail.com; 4 Cardiac Surgery Unit, Tor Vergata University, 00133 Rome, Italy; claudia.altieri@ptvonline.it

**Keywords:** aortic valve stenosis, aortic valve replacement, sutureless aortic prostheses

## Abstract

**Objective:** The aim of this study is to analyze the effects of sutureless aortic valve bioprosthesis implantation compared with stented conventional bioprosthesis in patients with severe aortic stenosis. This is a propensity matching institutional study. **Materials and Methods**: We compared 37 patients who underwent aortic valve replacement with Carpentier Edwards Perimount implantation (group 1) with 37 patients with sutureless Perceval S implanted (group 2). Preoperative, intraoperative, and postoperative parameters were studied. **Results:** The cross-clamp time, the mechanical ventilation times, the intensive care unit, and the hospital stay were significantly shorter in group 2 than in group 1 (*p*-value < 0.001). The cardio-pulmonary bypass time was 74 [45, 201] minutes in group 2 and 82 [48, 654] minutes in group 1 (*p*-value = 0.113). The postoperative mean gradients were 13 [6, 44] mmHg in group 2 and 14 [6, 19] mmHg in group 1 (*p*-value 0.285), and the effective orifice areas in these two groups were 1.5 ± 0.18 cm^2^ vs. 1.1 ± 0.4 cm^2^ (*p =* 0.002). The percentage of minimally invasive approach was higher in group 2 than in group 1. The echocardiographic follow-up analysis showed that the mean and maximum gradients with a sutureless prosthesis implant were lower than that of a traditional prosthesis, although this difference was not statistically significant. **Conclusions:** The Perceval S valve seems to be an effective alternative solution for biological valve implantation with good hemodynamic characteristics as compared with Carpentier Edwards Perimount prosthesis, providing shorter ischemic and extracorporeal circulation time and better postoperative recovery. Perceval S valve implantation facilitates the minimally invasive approach.

## 1. Introduction

Aortic valve (AV) stenosis is the most frequent cardiac valve pathology in the western world, with a prevalence of 3% over the age of 75 years [1]. The incidence of AV stenosis is growing, as a reflection of the rapid ageing of the population [2]. Multiple studies have showed the beneficial effects of aortic valve replacement (SAVR) with regard to improvement in quality of life and physical performance in the majority of symptomatic patients [3]. However, the treatment of aortic valve pathology has been constantly evolving for the last two decades with an increase in the use of transcatheter aortic valve replacement (TAVR) from the high toward the low-risk patients [4]. As TAVR has become more established, newer surgical prostheses have been developed with a variety of anchoring systems that do not rely solely on sutures to hold the valve in the appropriate location [5]. This new family of aortic prosthesis is called “sutureless prosthesis”. By avoiding placement and tying of annular sutures, sutureless aortic prosthesis significantly reduces operative and, more importantly, ischemic times and may improve outcomes [6,7,8]. Therefore, a sutureless aortic prosthesis should be considered in order to minimize operative times and improve outcomes in intermediate and high-risk patients in whom a long bypass run would be detrimental and in those undergoing complex combined procedures. On the other hand, with its collapsible design and easily handling, sutureless prosthesis facilitates a minimally invasive approach that has been shown to be safe and effective as the full sternotomy for AVR [9,10,11,12]. Accordingly, we report our experience that confirmed what has already been demonstrated by other authors. In addition, our analysis assessed the advantages of sutureless aortic bioprosthesis implantation in younger and lower-risk populations than the other studies.

## 2. Materials and Methods

### 2.1. Study Population

This was a retrospective multicenter study. We collected data of patients who underwent surgical aortic valve replacement (SAVR) from January 2022 to January 2023 and compared those who underwent SAVR with Carpentier Edwards Perimount (Edwards, Irvine, CA, USA) implantation (group 1) with those who received a sutureless Perceval S (CORCYM Srl, Saluggia, Italy) (group 2). A careful medical history was collected for all patients, and a series of preoperative clinical variables were evaluated. A quantification of the preoperative risk was made using the logistic Euroscore and Euroscore II. All patients underwent aortic valve replacement in extracorporeal circulation using either a traditional approach (median longitudinal sternotomy) or a minimally invasive approach. For sutureless prosthesis, we favored a right anterior mini-thoracotomy approach as we believe it is more favorable than a partial sternotomy and provides direct access to the aortic valve with beneficial effects for the patient. The Perceval implantation technique [13] and the minimally invasive approach were previously described [14,15,16]. The following intraoperative variables were evaluated: urgency/emergency, other concomitant cardiac or thoracic surgery, extracorporeal circulation time, aortic clamping time, repeated aortic clamping, migration, and valve malposition evaluated by transesophageal echocardiography. The following postoperative variables were evaluated: mechanical ventilation time, cardiac tamponade and postoperative bleeding requiring surgical revision, low cardiac output syndrome requiring high doses of inotropes and ventricular mechanical assistance (extra-corporeal membrane oxygenation (ECMO) and intra-aortic balloon pump (IABP)), heart attack postoperative, postoperative pacemaker (PMK) implantation, renal failure (acute renal failure and the need for dialysis), respiratory failure, intestinal ischemia, cerebrovascular complications (transient ischemic attack (TIA), stroke, and delirium), wound dehiscence, infectious complications, and platelet count. Both the intensive care stay (days) and in-hospital stay (days) were calculated. All patients underwent post-operative echocardiography to evaluate the following parameters: mean and peak gradient of the implanted prosthesis, effective orifice area (EOA) and indexed effective orifice area (iEOA) of the implanted prosthesis, ventricular size and function, presence of periprosthetic leak, and residual aortic insufficiency. Ultrasound examination and measurement were performed following published guidelines from the American Society of Echocardiography and other societies [17]. We adopted the threshold of an iEOA ≤ 0.85 cm^2^/m^2^ to define AV patient prosthesis mismatch (PPM), with values between 0.65 and 0.85 cm^2^/m^2^ as moderate PPM and <0.65 cm^2^/m^2^ as severe PPM [18]. An analysis of operative, hospital, and 30-day mortality has been conducted. Finally, we performed a clinical and echocardiographic follow-up of the patients under study by evaluating the following variables: mean and maximum gradient of the prosthesis, effective orifice area, and left ventricle function.

### 2.2. Statistical Analysis

To reduce the effect of selection bias and potential confounding factors, patients treated a stented prosthesis valve were matched with patients treated with a sutureless prosthesis valve by developing a propensity score matching. To estimate the propensity score for a particular sutureless patient, a logistic regression model was used. Variables involved in logistic regression model to generate the propensity score were age, sex, body max index, hypertension, diabetes, smoke, NYHA, mitral stenosis, tricuspid regurgitation, left ventricle end diastolic diameter, pulmonary hypertension, coronary artery disease, chronic lung disease, previous cardiac surgery, logistic Euroscore, Euroscore II, and preoperative platelets (PLT) count. We used 5 to 1 digit matching to identify the matched patients. Continuous data are presented as median and lower/upper, and categorical data are expressed as percentages and absolute values. The normality of the distribution of quantitative data was assessed using the Shapiro–Wilk test. The differences between the two groups of patients were calculated using the Student’s t-test, Welch test, or Wilcoxon rank sum test, as appropriate, for quantitative variables and the Pearson X2 test or exact Fisher test for qualitative variables. Variables were checked for normality and homoscedasticity, and the assumptions were accepted when *p* > 0.05. STATA 13.1 software (Stata Corp, College Station, TX, USA) was used for data analysis; a value of *p*-value < 0.05 was considered statistically significant.

## 3. Results

### 3.1. In-Hospital Data

Demographic, preoperative clinical, and echocardiographic characteristics of our study population (37 patients implanted with a Carpentier Edwards Perimount, group 1, and 37 with the Perceval valve, group 2) are reported in Table 1 and Table 2.

A propensity score matching (1:1) was performed to control selection bias; as a result of nonrandom assignment to the groups, we obtained 27 matched pairs with the same propensity score. Demographic, preoperative clinical, and echocardiographic characteristics of all matched pairs are reported in Table 3 and Table 4. No single value was statistically significant after matching.

As shown in Table 5 and Table 6, the cross-clamp times, the mechanical ventilation times, and the ICU and hospital stays were significantly shorter in group 2 than in group 1 (*p*-value < 0.001). The median cardio-pulmonary bypass time was 74 [45, 201] minutes in group 2 and 82 [48, 654] minutes in group 1 (*p*-value = 0.113). The median peak postoperative gradients were 22 [12, 45] mmHg in group 2 and 23 [11, 42] mmHg in group 1 (*p*-value 0.456). The mean postoperative effective orifice areas in these two groups were 1.5 ± 0.18 cm^2^ vs. 1.1 ± 0.4 cm^2^ (*p* = 0.002). The prosthetic valve sizes in group 1 were as follows: 19 mm in 4 cases (18.81%), 21 mm in 15 cases (40.54%), 23 mm in 14 cases (37.83%), and 25 mm in 4 cases (10.81%). The prosthetic valve sizes in group 2 were as follows: Perceval S in 7 cases (18.91%), Perceval M in 5 cases (13.51%), Perceval L in 11 cases (29.72%), and Perceval XL in 14 cases (37.83%). No differences were found in terms of operative, in-hospital and 30-days mortality. The percentage of minimally invasive approach was higher in group 2 than in group 1.

### 3.2. Data at Follow-Up

The mean follow-up was 36 ± 3 months. The echocardiographic follow-up analysis, performed in our out clinic patients by our cardiologist, showed that the mean and peak gradients of a sutureless prosthesis were lower than those of a traditional prosthesis, although this difference was not statistically significant.

## 4. Discussion

**Figure 1:**
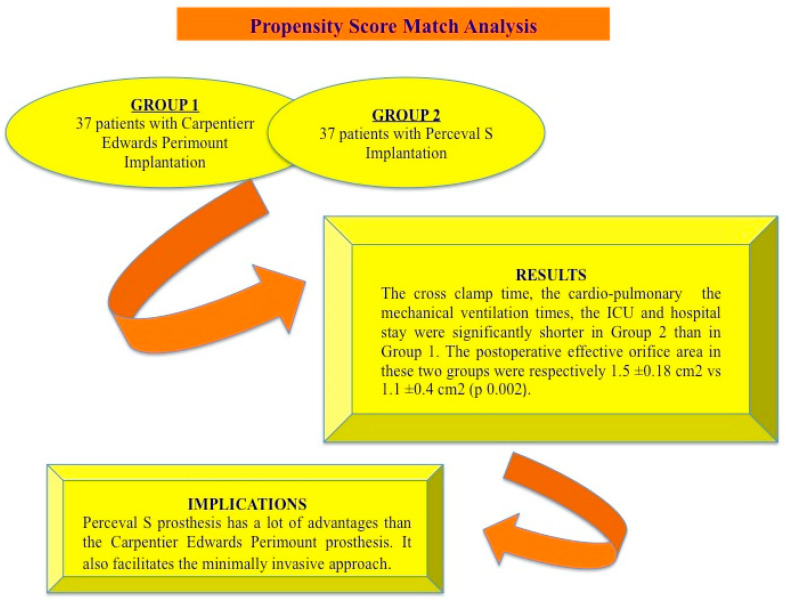
A summary of the advantages of sutureless bioprosthetic aortic valve: the Perceval S valve implantation seems to be an interesting biological valve with good hemodynamic characteristics as compared with the Carpentier Edwards Perimount prosthesis.

Our study was conducted with the aim to assess the effects of sutureless aortic valve bioprosthesis implantation compared with stented conventional bioprosthesis in patients with severe aortic stenosis through a retrospective review of our data. To reduce the bias related to retrospective observational comparison, a propensity score analysis was performed (Figure 1).

There are several considerations when looking at the outcomes of this propensity matched analysis. The age and the logistic Euroscore of our study population showed that in our center, patients undergoing AVR with biological prostheses, either sutureless or conventional, are becoming younger and at lower risk. In particular, in the group of patients that received a sutureless Perceval S prosthesis, the mean age was 71.1 ± 5.6 age, and the mean Logistic Euroscore was 3.7%. These values were lower than the data reported in the Sutureless and Rapid Deployment International Registry [19]. This finding was consistent with the current national trend in conventional AVR described both in the German and in the French registries [20,21]. Several reasons may explain this trend. First, there is dramatic growth in the transcatheter aortic valve implantation (TAVI) in older adult and higher-risk patients. Second, the trend supported the biological valves in younger and low-risk patients. Our analysis demonstrated several advantages of using Perceval prosthesis over conventional prostheses. In accordance with the literature data [22,23], in our study, the use of sutureless valves Perceval S prostheses was correlated to a shorter aortic cross-clamping time and extracorporeal circulation time, which have been shown to be strong independent predictors of postoperative morbidity and mortality. In particular, in a large retrospective analysis of approximately 1.000 patients undergoing surgical AVR, Ranucci et al. [24] reported an increased risk of 1.4% per 1 min increasing of aortic cross-clamp time. In our series, the cardiopulmonary bypass time (median 74 min) was similar to those reported in other studies on sutureless prosthetic valves; however, the cross-clamp time was shorter (44 min) than the cross-clamp time reported in other studies. In particular, D’Onofrio et al. [25] in a retrospective multicenter study reported a cardiopulmonary bypass time of 75 min and a cross-clamp time of 52 min. In the real-world German Aortic Valve Registry [26], the cardiopulmonary bypass time and the cross-clamp time during Perceval S implantation were 59 ± 21 min and 34 ± 15 min. In the Sutureless and Rapid Deployment International Registry (SURD-IR), Beretta et al. reported a cardiopulmonary bypass time of 63 (47–84) minutes and a cross-clamp time of 39.5 (29–53) minutes in isolated AVR using Perceval S prostheses [19]. In all cases, the surgical timings were consistently shorter than those reported in the STS database for conventional isolated surgical AVR (cross-clamp time, 78 min, and cardiopulmonary bypass time, 106 min), despite a large number of patients receiving a Perceval valve in the above-mentioned study, which was implanted through a minimally invasive approach. This confirms the time-sparing property of these valves when compared with conventional bioprostheses [27]. The time-sparing property of these devices becomes very important as well in combined operations [28], where the time spent with the aortic valve may be significantly reduced, and this was also clearly confirmed by our population in which nearly 40% of patients underwent associated procedures. In addition, according to our results the AVR with Perceval S prostheses has been proven to be hemodynamically advantageous compared with conventional AVR due to the absence of a suture ring with a larger effective orifice area, resulting in lower mean and peak transvalvular gradients as already assessed in other clinical and in vitro studies [29,30,31,32,33]. In particular, the postoperative mean gradients were 13 mmHg in patients who underwent AVR with sutureless Perceval S prostheses and 14 mmHg in patients who underwent AVR with conventional prostheses (*p*-value 0.285). The statistical nonsignificance might be related to the small sample size. On the other hand, the postoperative effective orifice areas in these two groups were 1.5 ± 0.18 cm^2^ vs. 1.1 ± 0.4 cm^2^ (*p =* 0.002). This could be explained by the way in which the sutureless valve is implanted. In fact, the balloon dilatation performed at the end of the release of the prosthesis helps to fully expand the inflow ring with a slight impact on the ventricular outflow tract. Probably, the presence of the very thin stent allows the valve to act like a stentless valve. Moreover, thanks to the self-expandable design, the internal diameter can adapt to the patient’s annulus with a benefit in fluid dynamic performance, contrary to what happens in conventional stented valves in which each size has a fixed internal diameter. To achieve the best hemodynamic performance, the Perceval S valve should not be oversized, to avoid potential issues related to stent folding, para-prosthetic leaks, and elevated gradient due to the incomplete valve expansion. These effects described above could also explain why, unlike our study, many authors highlighted an increase in pacemaker implantation in the sutureless group [7].

Another important benefit associated to the Perceval implantation is the shorter intubation time (4.3 h Perceval group vs. 9 h Perimount group, *p*-value <0.001) and consequently an early weaning from mechanical ventilation in patients treated with sutureless prostheses. These data are consistent with what is reported in the literature with benefits that affect the primary and secondary outcomes [34]. In addition, the observed difference in the hospital length of stay between patients treated with Perceval and conventional prostheses is an important area of comment. Indeed, reducing the hospital stay and shortening patient recovery should be considered key elements to evaluate the results of contemporary valve interventions, mainly in patients undergoing minimally invasive procedures. On the other hand, according to our clinical practice, the Perceval aortic valve offers the opportunity to make minimally invasive implantation easier and faster than if using another type of bioprosthetic valve. Consequently, we performed a minimally invasive approach in about 60% of patients who underwent Perceval valve implantation. In particular, we preferred a right anterior thoracotomy in accordance with the results of the Sutureless and Rapid Deployment Aortic Valve Replacement International Registry [14]. These results, in fact, showed that full sternotomy resulted in a higher rate of acute kidney injury; the anterior lateral thoracotomy had a lower stroke rate than ministernotomy and a shorter hospital stay than all other accesses. An important issue highlighted in our clinical practice is also the higher incidence of thrombocytopenia in patients who received sutureless implants compared with those who had Perimount implants. A decrease in platelets count after biological valve implantation compared with the mechanical valve has already been described in the literature [35]. In this specific case it could be explained by the type of treatment of the sutureless valve, which would seem to be not dissimilar to the Sorin Freedom SOLO prosthesis (same family, same treatment, and same problem). In the majority of the cases, we assisted in a rapid and progressive recovery of the platelet count a few days after Perceval S implantation without clinical consequences. An interesting explanation of the thrombocytopenia is the one reported in a recent pilot observational analysis using imaging and three-dimensional modeling by Aljalloud et al. [36] According these authors the Perceval bioprosthesis deformation during the implantation lead to fluttering, a reduction in the cusps’ mobility. This condition could potentially result in fibrosis as well as increased transvalvular pressure gradients and might be the cause for the increase in lactate dehydrogenase and the decrease in platelet count. Accordingly, we thought that a valve implantation technique as meticulous and precise as possible can reduce the risk of stent deformation and the incidence of thrombocytopenia. Finally, as reported in a recent metanalysis [7], we noted a higher risk of pacemaker implantation in patients with Perceval sutureless AVR. Previous branch block or atrioventricular block, age, and larger valve annulus size and calcifications are risk factors for pacemaker implantation after AVR. Surgical implantation technique may also contribute to atrioventricular conduction disorders. Accurate annulus decalcification, positioning of guide suture, minimizing the traction on the valve commissures, and the balloon pressure may reduce the rate of pacemaker implantation after sutureless AVR.

## 5. Conclusions

The Perceval S valve implantation seems to be an effective alternative solution for biological valve implantation with good hemodynamic characteristics as compared with Carpentier Edwards Perimount prosthesis providing shorter ischemic time, shorter extracorporeal circulation time, and better postoperative recovery. In addition, the Perceval S valve implantation facilitates the minimally invasive approach. In our opinion, the Perceval S valve prosthesis could be safely and effectively implanted in increasingly younger and lower-risk patients.

## 6. Study Limitation

The main limitation of the study was the small sample size. We know that the volume of patients is not powerful enough. Our purpose was to report our experience with sutureless prosthetic valves and compare our data with those already described in the literature. Another limitation of the study was the retrospective analysis. The statistical nonsignificance might be related to the small sample size. It is evident that this is not a randomized trial. For this reason, to reduce the effect of selection bias and potential confounding factors, stented prosthetic valve patients were matched with sutureless prosthetic valve patients by developing a propensity score matching.

## Figures and Tables

**Table 1:** Demographic and preoperative clinical characteristics of the two groups of patients (group 1, Carpentier Edwards Perimount, and group 2, Perceval S) before propensity match score analysis.

Variables	Group 1 (37 Patients)	Group 2 (37 Patients)	*p*-Value
Age (years), mean ± SD	71.1 ± 5.6	71.1 ± 5.6	1.000
Sex, female	17 (45.9%)	18 (48.6%)	0.816
Height (cm), mean ± SD	164.2 ± 7.3	160 ± 9.7	0.037
Weight (kg), mean ± SD	75.1 ± 13.1	70.1 ± 12	0.089
BMI	27.8 ± 4.1	27.5 ± 4.7	0.759
Hypertension	33 (89.2%)	32 (86.5%)	0.722
Diabetes	4 (10.8%)	9 (24.3%)	0.221
Dyslipidaemia	24 (64.9%)	19 (51.4%)	0.239
Tobacco use	4 (10.8%)	9 (24.3%)	0.221
Preop atrial fibrillation	5 (13.5%)	2 (5.4%)	0.430
NYHA class			0.203
I	1 (2.7%)	5 (13.5%)	
II	16 (43.2%)	16 (43.2%)	
III	20 (54.1%)	15 (40.5%)	
IV	0 (0%)	1 (2.7%)	
Coronary artery disease	14 (37.8%)	9 (24.3%)	0.209
Previous MI	2 (5.4%)	5 (13.5%)	0.430
PCI/Stent	1 (2.7%)	3 (8.1%)	0.615
Peripheral vascular disease	0 (0%)	3 (8.1%)	0.240
Cerebrovascular disease	8 (21.6%)	3 (8.1%)	0.190
Previous stroke	1 (2.7%)	1 (2.7%)	1.000
Previous TIA	1 (2.7%)	1 (2.7%)	1.000
Significant carotid artery disease	5 (13.5%)	3 (8.1%)	0.711
Renal insufficiency	0 (0%)	6 (16.2%)	0.025
Creatinine (mg/dL), median [IQR]	0.9 [0.6, 2.9]	1 [0.5, 2.1]	0.411
Chronic lung disease	6 (16.2%)	6 (16.2%)	1.000
Previous cardiac surgery	3 (8.1%)	6 (16.2%)	0.479
Previous aortic valve surgery	1 (2.7%)	5 (13.5%)	0.199
Previous other valvular surgery	0 (0%)	0 (0%)	-
Previous CABG	0 (0%)	0 (0%)	-
Previous thoracic aorta surgery	0 (0%)	0 (0%)	-
LogisticEuroSCORE, median [IQR]	2.9 [1.1, 11]	4.5 [1.1, 39.3]	0.035
EuroSCORE II, median [IQR]	1.2 [0.7, 3.4]	1.4 [0.6, 13.7]	0.678
Preoperative platelet count (mil/μL) median [IQR]	210,000 [115,000, 484,000]	200,500 [88,000, 326,000]	0.427

SD—standard deviation; BMI—body mass index; NYHA—New York Heart Association; MI—myocardial infarction; PCI—percutaneous coronary intervention; TIA—transient ischemic attack; CABG—coronary artery bypass graft; IQR—interquartile range.

**Table 2:** Echocardiographic data of the two groups of patients (group 1, Carpentier Edwards Perimount, and group 2, Perceval S) before propensity match score analysis.

Variables	Group 1 (37 Patients)	Group 2 (37 Patients)	*p*-Value
Bicuspid aortic valve	5 (13.5%)	8 (21.6%)	0.543
Aortic valve stenosis	37 (100%)	35 (94.6%)	0.493
Aortic valve regurgitation	26 (70.3%)	17 (45.9%)	0.034
Mixed aortic valve disease	-	-	-
Mitral regurgitation			0.020
0	23 (62.2%)	11 (29.7%)	
1	12 (32.4%)	22 (59.5%)	
2	2 (5.4%)	3 (8.1%)	
3	0 (0%)	1 (2.7%)	
Mitral stenosis			0.240
0	36 (97.3%)	33 (89.2%)	
1	0 (0%)	3 (8.1%)	
2	1 (2.7%)	1 (2.7%)	
Tricuspid regurgitation			0.359
0	22 (59.5%)	15 (40.5%)	
1	14 (37.8%)	20 (54.1%)	
2	1 (2.7%)	2 (5.4%)	
AVA (cm^2^), mean ± SD	0.7 ± 0.2	0.8 ± 0.2	0.293
AVAi (cm^2^/m^2^), median [IQR]	0.4 [0.2, 1]	0.4 [0.2, 0.7]	0.550
Peak aortic valve gradient (mmHg), median [IQR]	70.5 [52, 155]	71 [13, 165]	0.749
Mean aortic valve gradient (mmHg), median [IQR]	44 [34, 92]	45 [6.5, 102]	0.978
LVEF%, median [IQR]	60 [42, 70]	60 [43, 73]	0.533
Pulmonary hypertension	6 (16.2%)	5 (13.5%)	1.000

AVA—aortic valve area; AVAi—aortic valve area index; LVEF—left ventricular ejection fraction; SD—standard deviation; IQR—interquartile range.

**Table 3:** Demographic and preoperative clinical characteristics of the two groups of patients (group 1, Carpentier Edwards Perimount, and group 2, Perceval S) after propensity match score analysis.

Variables	Group 1 (27 Patients)	Group 2 (27 Patients)	*p*-Value
Age (years), median [IQR]	69.4 [63.8, 75]	70.5 [65.3, 75.7]	0.454
Sex, female	11 (40.7%)	12 (44.4%)	0.783
Height (cm), median [IQR]	165.6 [158.2, 173]	161.2 [151.5, 170.9]	0.066
Weight (kg), median [IQR]	76.6 [63.4, 89.8]	71.9 [59.5, 84.3]	0.178
BMI, median [IQR]	27.8 [23.9, 31.7]	27.7 [23, 32.4]	0.925
Hypertension	23 (85.2%)	24 (88.9%)	1.000
Diabetes	3 (11.1%)	6 (22.2%)	0.467
Dyslipidemia	19 (70.4%)	14 (51.9%)	0.163
Tabacco use	4 (14.8%)	7 (25.9%)	0.501
Atrial fibrillation	3 (11.1%)	1 (3.7%)	0.610
NYHA class			0.227
I	1 (3.7%)	4 (14.8%)	
II	11 (40.7%)	13 (48.1%)	
III	15 (55.6%)	10 (37%)	
IV	0 (0%)	1 (3.7%)	
	4 (14.8%)	7 (25.9%)	0.501
Active endocarditis	0 (0%)	0 (0%)	-
Coronary artery disease	10 (37%)	8 (29.6%)	0.564
Previous MI	2 (7.4%)	4 (14.8%)	0.669
PCIStent	1 (3.7%)	3 (11.1%)	0.610
Peripheral vascular disease	0 (0%)	0 (0%)	0.240
Cerebrovascular disease	5 (18.5%)	1 (3.7%)	0.192
Previous stroke	1 (3.7%)	1 (3.7%)	1.000
Previous TIA	1 (3.7%)	0 (0%)	1.000
Carotid artery disease	2 (7.4%)	0 (0%)	0.491
Renal insufficiency	0 (0%)	3 (11.1%)	0.236
Creatinine (mg/dL), median [IQR]	0.9 [0.6, 1.9]	0.9 [0.5, 1.5]	0.993
Chronic lung disease	4 (14.8%)	3 (11.1%)	1.000
Previous cardiac surgery	2 (7.4%)	4 (14.8%)	0.669
Previous aortic valve surgery	1 (3.7%)	3 (11.1%)	0.610
LogisticEuroSCORE, median [IQR]	2.9 [1.1, 11]	3.7 [1.1, 33.8]	0.197
EuroSCORE II, median [IQR]	1.2 [0.7, 3.4]	1.2 [0.6, 13.7]	0.802
Preoperative platelets count (mil/μL), median [IQR]	210,000 [132,000, 484,000]	230,000 [150,000, 326,000]	0.716

BMI—body mass index; PMK—pacemaker; NYHA—New York Heart Association; CCS—Canadian Cardiovascular Society; MI—myocardial infarction; PCI—percutaneous coronary intervention; TIA—transient ischemic attack; CABG—coronary artery bypass graft; IQR—interquartile range.

**Table 4:** Echocardiographic data of the two groups of patients (group 1, Carpentier Edwards Perimount implanted, and group 2, Perceval S) after propensity match score analysis.

Variables	Group 1 (27 Patients)	Group 2 (27 Patients)	*p*-Value
Bicuspid aortic valve	4 (14.8%)	7 (25.9%)	0.501
Aortic valve stenosis	27 (100%)	26 (96.3%)	1.000
Aortic valve regurgitation	17 (63%)	11 (40.7%)	0.102
Mixed aortic valve disease	-	-	-
Mitral regurgitation			0.073
0	16 (59.3%)	8 (29.6%)	
1	9 (33.3%)	17 (63%)	
2	2 (7.4%)	2 (7.4%)	
3	0 (0%)	0 (0%)	
Mitral stenosis			0.236
0	26 (96.3%)	23 (85.2%)	
1	0 (0%)	3 (11.1%)	
2	1 (3.7%)	1 (3.7%)	
Tricuspid regurgitation			0.215
0	16 (59.3%)	10 (37%)	
1	10 (37%)	16 (59.3%)	
2	1 (3.7%)	1 (3.7%)	
AVA (cm^2^), median [IQR]	0.7 [0.5, 0.9]	0.8 [0.6, 1.1]	0.389
AVAi (cm^2^/m^2^), median [IQR]	0.4 [0.2, 1]	0.4 [0.3, 0.7]	0.439
Peak gradient (mmHg), median [IQR]	76 [54, 155]	72 [17, 151]	0.762
Mean aortic valve gradient (mmHg), median [IQR]	46 [34, 92]	46 [24, 97]	0.852
LVEF %, median [IQR]	60 [42, 67.6]	61 [45, 70]	0.456
Pulmonary hypertension	4 (14.8%)	2 (7.4%)	0.669

AVA—aortic valve area; AVAi—aortic valve area index; LVEF—left ventricular ejection fraction; IQR—interquartile range.

**Table 5:** Intra-operative data of the propensity-matched population.

Variables	Group 1 (27 Patients)	Group 2 (27 Patients)	*p*-Value
Urgent/emergent status	1 (3.7%)	0 (0%)	1.000
Full sternotomy	22 (81.5%)	11 (40.7%)	0.005
Ministernotomy	4 (14.8%)	4 (14.8%)	1.000
Minithoracotomy	1 (3.7%)	11 (40.7%)	0.002
Conversion to full sternotomy	0 (0%)	0 (0%)	-
Concomitant procedures	7 (25.9%)	11 (40.7%)	0.387
Concomitant thoracic aorta surgery	1 (3.7%)	1 (3.7%)	1.000
Concomitant septal myectomy	0 (0%)	6 (22.2%)	0.023
CPB time (minute), median [IQR]	82 [48, 654]	74 [45, 201]	0.113
Cross-clamp time (minute), median [IQR]	62 [41, 145]	44 [30, 116]	0.002
Valve malpositioning migration	0 (0%)	0 (0%)	-
Repeated cross clamping	0 (0%)	1 (3.7%)	1.000
Transfusion			0.093
0	20 (74.1%)	13 (48.1%)	
1	7 (25.9%)	13 (48.1%)	
2	0 (0%)	1 (3.7%)	

CPB—cardio pulmonary bypass time.

**Table 6:** Post-operative outcomes and follow-up data of the propensity-matched population.

Variables	Group 1 (27 Patients)	Group 2 (27 Patients)	*p*-Value
**Post-operative variables**			
Aortic prosthesis peak gradient (mmHg), median [IQR]	23 [11, 42]	22 [12, 45]	0.456
Aortic prosthesis mean gradient (mmHg), median [IQR]	14 [6, 19]	13 [6, 44]	0.285
LVEF %, median [IQR]	58.7 [50, 65]	60 [48, 66]	0.541
Aortic regurgitation	1 (3.7%)	0 (0%)	1.000
Paravalvular leak	0 (0%)	0 (0%)	-
Postoperative myocardial infarction	0 (0%)	2 (7.4%)	0.491
Cardiac tamponade	0 (0%)	0 (0%)	-
Cardiac failure	0 (0%)	0 (0%)	-
Delirium	0 (0%)	2 (7.4%)	0.491
Stroke	0 (0%)	0 (0%)	-
TIA	0 (0%)	0 (0%)	-
Low platelet count	3 (11.1%)	20 (74.1%)	<0.001
Bleeding requiring chest reopening	1 (3.7%)	0 (0%)	1.000
AKI	0 (0%)	2 (7.4%)	0.491
Permanent PMK implantation	2 (7.4%)	0 (0%)	0.491
Atrial fibrillation	13 (48.1%)	12 (44.4%)	1.000
Respiratory failure	1 (3.7%)	1 (3.7%)	1.000
Ventilation time	9 [3, 48]	4.3 [0, 62.2]	<0.001
ICU stay (hours), median [IQR]	24.1 [11.2, 184.7]	24 [24, 168]	0.129
Hospital stay (days), median [IQR]	10 [6, 75]	9 [4, 19]	0.511
Hospital mortality	0 (0%)	0 (0%)	-
30-day mortality	0 (0%)	0 (0%)	-
Operative mortality	0 (0%)	0 (0%)	-
**Follow-Up Variables**			
Aortic valve prosthesis peak gradient (mmHg), median [IQR]	22 [10, 41]	21 [11, 44]	0.642
Aortic valve prosthesis mean gradient (mmHg), median [IQR]	15 [7, 20]	12.1 [5, 43]	0.211
EOA, median [IQR]	1.1 [0.8, 1.3]	1.5 [1.2, 1.7]	0.002
LVEF%, median [IQR]	56 [50, 60]	57 [51, 61]	1.000

LVEF%—left ventricle ejection fraction %; TIA—transient ischemic attack; AKI—acute kidney injury; PMK—pacemaker; ICU—intensive care unit; EOA—effective orifice area.

## Data Availability

The raw data supporting the conclusions of this article will be made available by the authors on request.

## Abbreviations

The following abbreviations are used in this manuscript: SAVR, surgical aortic valve replacement; TAVR, transcatheter aortic valve replacement; AVR, aortic valve replacement; ICU, intensive care unit; ECMO, extracorporeal membrane oxygenation; IABP, intra-aortic ballon pump; TIA, transient ischemic attack; PMK, pacemaker; EOA, effective orifice area; iEOA, index effective orifice area; PPM, patient–prosthesis mismatch; NYHA, New York Heart Association; PLT, platelet; TAVI, transcatheter aortic valve implantation; SURD-IR, Sutureless and Rapid Deployment International Registry.
